# Cocaine-associated plasma cell orificial mucositis (CAPCOM): An emerging entity

**DOI:** 10.1016/j.jdcr.2026.04.020

**Published:** 2026-04-21

**Authors:** Priya Patel Housley, Sophie Tillotson, Milbrey Parke, Kiran Motaparthi

**Affiliations:** a College of Medicine, University of Florida, Gainesville, Florida; b Department of Dermatology, College of Medicine, University of Florida, Gainesville, Florida

**Keywords:** ANCA-associated vasculitis, cocaine, cocaine-associated disorders, cocaine-associated plasma cell orificial mucositis (CAPCOM), levamisole, plasma cell mucositis

## Introduction

Cocaine-associated plasma cell orificial mucositis (CAPCOM), an emerging entity of increasing significance given the widespread use of cocaine, has been described recently and is thought to be a hypersensitivity reaction to a novel unidentified allergen in cocaine.^1^ Cocaine, along with its frequent adulterant levamisole, can cause a spectrum of cutaneous and mucosal disorders that may clinically and serologically mimic antineutrophil cytoplasmic antibody (ANCA)-associated vasculitis (AAV), risking unnecessary immunosuppression. CAPCOM is differentiated histologically by marked epidermal hyperplasia with a plasma cell-rich and often eosinophil-rich, infiltrate notably without vasculitis or granulomas.^2^^,^^3^ We present a series of 3 patients with clinical and pathologic features of CAPCOM and describe its relationship to and distinction from other cocaine spectrum disorders, including cocaine-induced midline destructive lesions (CIMDL), as well as AAV, namely granulomatosis with polyangiitis (GPA).

## Case 1

**Fig 1:**
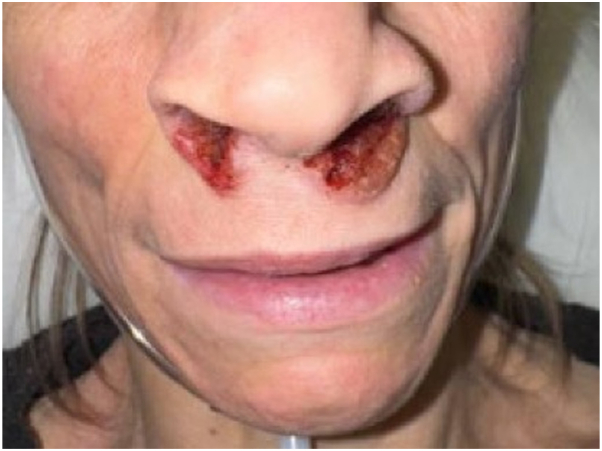
Case 1. Erythematous vegetative plaques with hemorrhagic crusting of the infranasal upper cutaneous lip.

**Fig 2:**
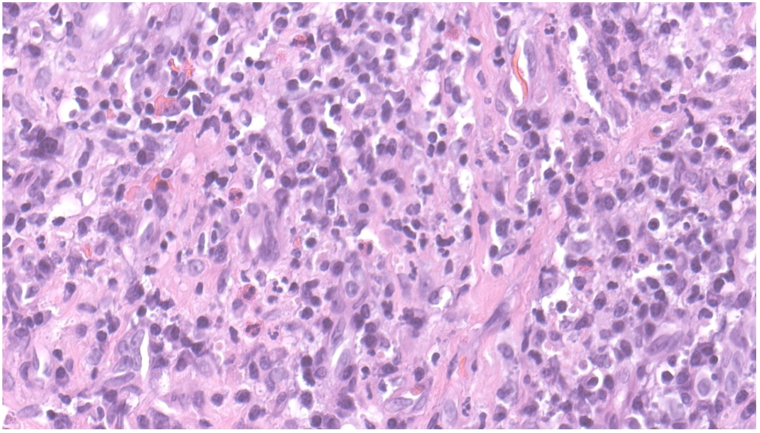
Case 1. Numerous plasma cells and eosinophils in dermis without granulomas or vasculitis (H&E, 400× magnification).

A 46-year-old woman with asthma presented with 1 month of progressive dyspnea and painful erythematous vegetative plaques with hemorrhagic crusting of the infranasal upper cutaneous lip (Fig 1). She reported frequent intranasal albuterol use and initially denied illicit drug use. A computed tomography (CT) of the chest demonstrated ground glass opacities. Workup included ANCA serologies and urine toxicology screening. Indirect immunofluorescence (IIF) demonstrated a cytoplasmic ANCA (c-ANCA) pattern (1:80) with anti-proteinase 3 (PR3) antibody positivity (407 U) and anti-myeloperoxidase (MPO) antibody negativity; urine toxicology was positive for cocaine metabolites. Laboratory evaluation demonstrated preserved renal function (BUN 18, creatinine 0.58). Punch biopsy of the upper cutaneous lip demonstrated epidermal hyperplasia and a dense mixed dermal inflammatory infiltrate rich in plasma cells with scattered eosinophils (Fig 2), without vasculitis or granulomatous inflammation.

At follow-up, she disclosed intranasal cocaine use, most recently 1 month prior to admission. As the clinicopathologic findings were most consistent with CAPCOM, rituximab therapy was deferred, and treatment included counseling on cocaine abstinence and a 6-week tapered course of prednisone, which was effective for the cutaneous and respiratory findings.

## Case 2

**Fig 3:**
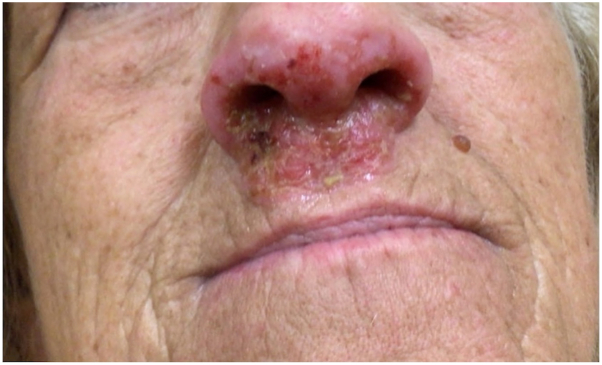
Case 2. Vegetative plaque with pustules involving the nares and upper cutaneous lip.

**Fig 4:**
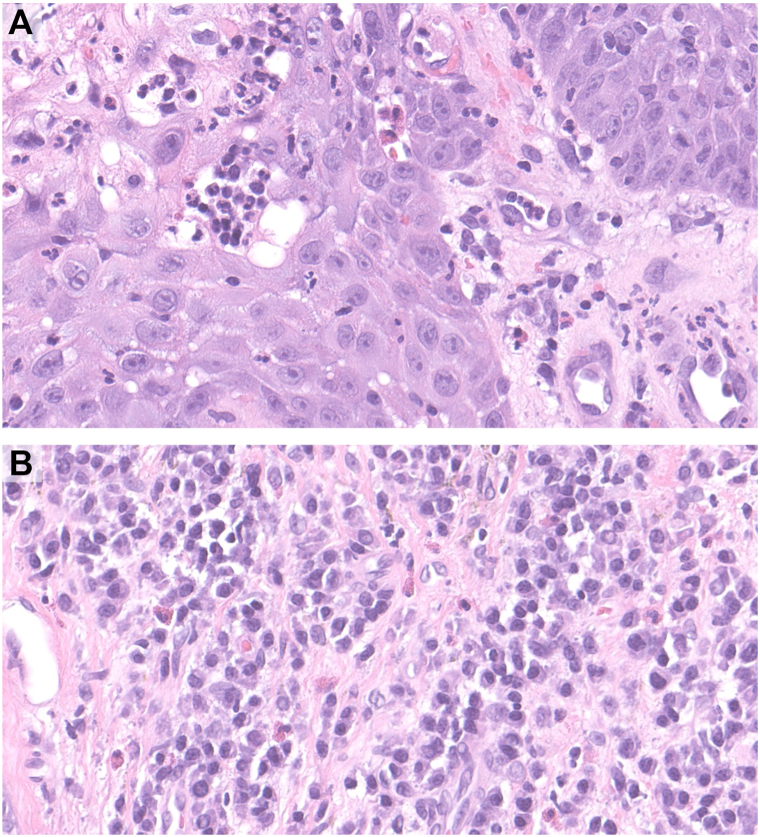
Case 2. **A,** Eosinophilic and neutrophilic spongiosis (H&E, 400× magnification). **B,** Numerous plasma cells and eosinophils in dermis, without vasculitis or granulomas (H&E, 400× magnification).

A 60-year-old woman presented with a 3-month history of a painful, vegetative plaque with pustules involving the nares and upper cutaneous lip (Fig 3) that was refractory to multiple antibiotics including doxycycline, cephalexin, and topical mupirocin. A punch biopsy was obtained, and histopathology showed eosinophilic spongiosis with a dense inflammatory infiltrate with numerous plasma cells, eosinophils, and neutrophils in the dermis (Fig 4); there was no evidence of vasculitis or granulomas. Direct immunofluorescence (DIF), IIF, and ELISA for desmoglein 1 and 3 and bullous pemphigoid antigens 1 and 2 were negative. Although ANCA testing by IIF was negative, ELISA demonstrated PR3 positivity with MPO negativity, raising suspicion for a cocaine-associated etiology. Systemic corticosteroids were initiated, with rapid resolution in findings and symptoms.

During follow-up, anamnesis confirmed cocaine nasal insufflation, most recently 1 month prior to lesion onset. Sinonasal CT imaging demonstrated mild septal change, but no destructive necrosis, arguing against a diagnosis of CIMDL. The constellation of isolated sinonasal findings, anti-PR3 positivity, and clinical resolution with cocaine abstinence and a 4-week prednisone taper supported a cocaine-associated etiology, with clinicopathologic findings most consistent with CAPCOM.

## Case 3

**Fig 5:**
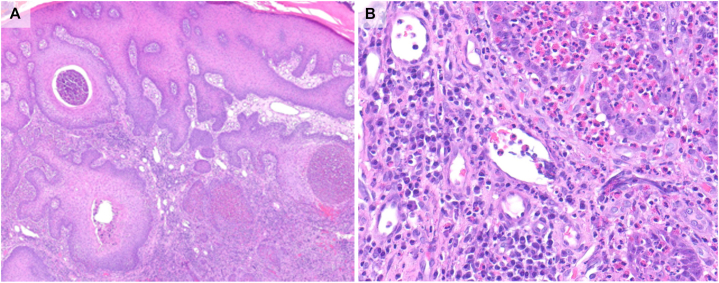
Case 3. **A,** Pseudoepitheliomatous hyperplasia with eosinophilic spongiosis including intraepidermal eosinophilic abscesses (H&E, 40× magnification). **B,** Numerous plasma cells and eosinophils in dermis (H&E, 400× magnification).

A 55-year-old man presented to an outside facility with vegetative plaques of the upper cutaneous lip and nasolabial folds. As urine toxicology was not obtained initially, and serology demonstrated c-ANCA positivity by IIF with PR3 positivity and MPO negativity, the patient was initially managed as GPA. Upon further investigation, histopathology was significant for pseudoepitheliomatous hyperplasia with dermal abscesses and a dense inflammatory infiltrate of numerous plasma cells, eosinophils, and neutrophils, without vasculitis or granulomas (Fig 5). Given the histopathologic findings and absence of systemic disease, the findings did not support AAV/GPA, and the disease was reclassified as CAPCOM.

## Discussion

Cocaine exposure, often in the setting of levamisole adulteration, is associated with a spectrum of mucocutaneous and sinonasal disorders including levamisole-associated vasculopathy/vasculitis, cocaine-associated neutrophilic dermatoses (including pyoderma gangrenosum-like disease), CIMDL, and the emerging entity of CAPCOM.^2^, ^3^, ^4^ As seen in this series, these disorders may closely mimic AAV, particularly GPA, both clinically with features such as ulceration, septal defects, pulmonary findings and serologically with ANCA positivity, creating risk for misdiagnosis and unnecessary long-term immunosuppression. Serologic distinction of cocaine-associated disorders from AAV can be challenging; in a series, 87.5% of patients with CIMDL were ANCA-positive, with 56% demonstrating PR3 positivity and the remainder MPO positivity.^5^ Furthermore, for the diagnosis of GPA, a c-ANCA/PR3 pattern has a specificity of 99.7% (range 99.4% to 99.8%); however, the positive predictive value has been shown to be 21.7% (range 9.7% to 41.9%), highlighting the importance of considering pre-test probability and the clinical context.^6^ Cocaine exposure may also result in pulmonary nodules and infiltrates due to levamisole exposure, closely simulating the respiratory disease of GPA.^7^ Differentiation from primary autoimmune disease is supported based on detection of ANCA directed against human neutrophil elastase (HNE) which, although highly specific for cocaine-associated etiologies, is not widely available for clinical use.^3^^,^^7^

The pathogenesis of cocaine-associated disorders provides context for the clinical and serological overlap with AAV. Cocaine, a potent vasoconstrictor, causes endothelial toxicity, while levamisole augments neutrophil activation. Together, exposure can promote formation of neutrophil extracellular traps (NETs); as in AAV, NETs induce ANCA production and directly induce vascular damage.^4^ However, in cocaine/levamisole etiologies, the NETs are coated in granules of multiple antigenic targets including HNE, PR3, MPO, cathepsin, lactoferrin, azurocidin, and/or catalase K.^3^^,^^4^ Furthermore, cocaine/levamisole activates c5b-9, the terminal complement complex, which leads to concurrent thrombosis in addition to vasculitis, a pattern not observed in AAV.^4^

In contrast to other cocaine/levamisole-associated disorders which are neutrophil driven and linked to c5b-9 formation, CAPCOM appears to reflect a hypersensitivity reaction to a novel unidentified cocaine adulterant, as evidenced by eosinophilia and elevated immunoglobulin E (IgE) in previously reported patients.^3^ Because vasculitis and granulomatous inflammation are not observed in CAPCOM, ANCA positivity may reflect circulating immune activation from cocaine exposure, rather than the pathogenesis of orificial lesions. Across the cases described here, the diagnosis of CAPCOM was supported by marked epidermal hyperplasia, variable intraepidermal pustules with eosinophils, and a brisk mixed dermal infiltrate with numerous eosinophils and plasma cells.

Within the spectrum of cocaine-associated disorders, summarized in Table I, overlapping or concurrent features may be observed. For instance, pyoderma gangrenosum-like ulcers associated with levamisole exposure, which demonstrate neutrophil-predominant infiltrate and vascular injury without granulomas, may be observed in tandem with palatal perforation (CIMDL) and pulmonary nodules.^6^ While CAPCOM and CIMDL are often concurrent findings with overlapping serologic findings, CIMDL demonstrates extensive necrosis due to ischemia and typically exhibits either vasculitis or thrombosis. In this series, CIMDL was initially considered in case 2; however, sinonasal imaging lacked destructive necrosis, and histopathology did not exhibit vasculitis or thrombosis, supporting classification as CAPCOM.

**Table I:** Spectrum of mucocutaneous disorders associated with cocaine insufflation

Disorder	Clinical presentation	Systemic involvement	Serology	Histopathologic features
Levamisole-associated vasculitis/vasculopathy^4^^,^^8^^,^∗	Inflammatory retiform purpura with predilection for acral sites including ears.	May include fever, arthritis, nephritis, heterogenous pulmonary disease, low complement, lymphopenia, and/or neutropenia.	ANCA positive (85%).^8^ Often p-ANCA pattern with multiple targets inc. anti-MPO. +ANA and APLs in most. +dsDNA.	LCV with or without thrombotic occlusion, affecting small and medium vessels. Fibrinoid necrosis and sparse inflammation with or without thrombotic occlusion. Pyoderma gangrenosum-like-pattern: neutrophilic dermatosis with vasculitis or vasculopathy.
Neutrophilic dermatoses^4^^,^^9^^,^∗	Multiple ulcers or vegetative plaques with predilection for the head, ears, and neck.	May include fever, night sweats, arthralgia, leukopenia, and/or renal involvement (commonly pauci-immune crescentic glomerulonephritis and membranous nephropathy).	Often p-ANCA pattern (71%) with antibody targets of anti-MPO and anti-HNE.^9^	Neutrophilic dermatosis with thrombotic vasculitis. or Granulomatous pattern resembling superficial granulomatous pyoderma without vasculitis
Cocaine-induced midline destructive lesions (CIMDL)^4^^,^^10^^,^^11^	Destructive sinonasal necrosis often leading to septal and/or palatal perforation.	Systemic findings typically absent.	p-ANCA pattern more often than c-ANCA. Antibody targets of anti-PR3 more often than anti-MPO, and anti-HNE (84%).^11^	Prominent necrosis with plasma-cell rich infiltrate and LCV with or without thrombosis of venules/arterioles (fibrin deposition or organizing intravascular granulation tissue). No granulomas.
Cocaine-associated plasma cell orificial mucositis (CAPCOM)^1^, ^2^, ^3^	Periorificial vegetative plaques or ulcers, commonly involving nares, upper cutaneous lip, and/or oral mucosa.	Systemic findings typically absent. May present with other cocaine-associated disorders.	c-ANCA more often than p-ANCA pattern. Multiple targets present, including anti-HNE.	Marked epidermal hyperplasia, variable intraepidermal pustules with eosinophils, and a brisk mixed dermal infiltrate with numerous eosinophils and plasma cells. No vasculitis or granulomas.

Information compiled from literature.^1^, ^2^, ^3^, ^4^^,^^8^, ^9^, ^10^, ^11^

*LCV*, Leukocytoclastic vasculitis.

∗ Associated with cocaine and/or cocaine adulterated with levamisole.

The histopathology of AAV may be supportive rather than diagnostic, and classic features may be absent. In a retrospective review, skin biopsies of AAV most commonly demonstrated leukocytoclastic vasculitis (LCV) (29%), followed by extravascular granulomas (22%), perivascular inflammation (18%), or nonspecific changes (17%), including spongiotic dermatitis patterns or changes of healing skin.^12^ The finding of LCV was noted to be subtle in some cases, and timing of the biopsy likely impacted diagnostic yield.^12^ Furthermore, the isolated histopathologic finding of LCV (small vessel vasculitis) is nonspecific and is also observed in cocaine-associated disorders, connective tissue diseases, and infections.^13^ Additionally, given that much of the data describing features of AAV is retrospective, unrecognized cocaine exposure may have confounded interpretation in some reported cases.

Our cases, together with previous literature, support consideration of cocaine-associated disorders for patients undergoing evaluation for AAV.^14^ Histopathology is central in the distinction, as cocaine-associated disorders tend to lack the combination of vasculitis and granulomas seen in GPA. CAPCOM presents with neither vasculitis nor granulomas, but instead a plasma-cell, and often eosinophil, rich infiltrate. Additionally, atypical ANCA patterns with multiple targets, especially HNE, may raise suspicion for a cocaine-associated etiology over AAV. Because cocaine use is often not disclosed and may be uncovered after a prolonged clinical course and/or biopsy, as observed in our cohort, urine toxicology should routinely be performed for patients in whom AAV is being considered to reduce diagnostic delay and inappropriate immunosuppression. In a series of 7 patients, clinicians revised their diagnosis from GPA to CIMDL only after identification of cocaine metabolites.^15^ Of those patients, all were given inappropriate long-term immunosuppression including rituximab, high-dose corticosteroids, methotrexate, azathioprine, and mycophenolate mofetil.^15^

## Conflicts of interest

None disclosed.
